# Loss of CB1 receptors leads to differential age-related changes in reward-driven learning and memory

**DOI:** 10.3389/fnagi.2012.00034

**Published:** 2012-12-05

**Authors:** Onder Albayram, Andras Bilkei-Gorzo, Andreas Zimmer

**Affiliations:** Institute of Molecular Psychiatry, University of BonnBonn, Germany

**Keywords:** cannabinoid receptor, aging, emotional valence, motivation, spatial learning, mice

## Abstract

Previous studies have shown that cannabinoid 1 (CB1) receptor signaling dissociates between reward-associated and aversive memories. The influence of CB1 receptors on the aversion-driven spatial learning in the Morris water maze test is strongly age-dependent: mice with genetic deletion of CB1 receptors (Cnr1^−/−^) show superior learning when young but inferior learning when old compared to age-matched wild-type mice. Whether the reward-driven spatial learning is influenced in the same way by CB1 receptor signaling as the aversion-driven learning remains unclear. Thus, we examined the performance of Cn1^−/−^ and their wild-type littermates at ages of 2-, 5-, and 12-months-old in the eight-arm radial maze test—a reward-motivated model of spatial learning. Interestingly, 2-months-old Cnr1^−/−^ mice had a superior learning ability to wild-type mice. At the age of 5-months, Cnr1^−/−^ mice showed the same performance as the wild-type littermates. However, 12-months-old Cnr1^−/−^ mice showed significantly impaired performances in each parameter of the test. Accordingly, this study provides compelling support for our previous result that genetic deletion of CB1 receptor leads to early onset of age-related memory decline, similarly affecting both reward and aversion-driven learning.

## Introduction

Emotional arousal facilitates memory formation therefore memories about highly emotional situations are usually vivid, long-lasting, and resistant to extinction. The cannabinoid system influences the formation, extinction and reconsolidation of emotional memories but the latter two phases are dependent on the emotional significance of the memory. Increased endocannabinoid signaling either via inhibition of endocannabinoid uptake (Chhatwal and Ressler, 2007) or inhibition of anandamide degradation (Varvel et al., 2007) facilitates the extinction of fear-related memories whereas pharmacological or genetic blockade of cannabinoid 1 (CB1) receptors impairs fear memory extinction (Marsicano et al., 2002). The extinction of reward-related memories is not affected by pharmacological activation or blockade (Manwell et al., 2009) or by genetic deletion of CB1 receptors (Holter et al., 2005). The effect of cannabinoids on the reinstatement of fear- and reward-associated memories also differs. Activation of CB1 receptors blocks the reconsolidation of fear memories (Chhatwal and Ressler, 2007), whereas the reinstatement of reward-associated memories are decreased by CB1 antagonists (Ward et al., 2009). The dissociation between the effects of the cannabinoid system on fear and reward-associated memory was also shown in humans. Carriers of a minor gene variant of the fatty acid amide hydrolase, the degrading enzyme of the endogenous cannabinoid anandamide, show decreased threat-related reactivity in the amygdala but elevated reactivity to reward related signals in the ventral striatum compared to bearers of the major allele (Hariri et al., 2009). We have previously shown that the effect of CB1 receptor on spatial memory depends on the age of the animal: animals lacking CB1 receptors (Cnr1^−/−^) at the age of 6-weeks showed an improved performance in the Morris water maze test whereas 12-months-old Cnr1^−/−^ mice showed inferior performance (Albayram et al., 2011). The Morris water maze is one of the most widely used behavioral model to test hippocampus-dependent spatial learning and memory (Morris et al., 1982). This model is aversion-driven because being in water is aversive for mice and they try to escape to dryer territory (Akirav et al., 2001). Whether the effect of CB1 receptor on reward-motivated learning is also age-dependent is not known. Therefore we now asked whether the learning phenotype of Cnr1^−/−^ mice in the reward-driven eight-arm radial maze model also differs between the age groups.

## Materials and methods

### Animals

Experiments to test the consequence of a constitutive deletion of the CB1 receptor were carried out with group-housed male and female Cnr1^−/−^ and Cnr1^+/+^ littermates on a congenic C57BL6/J background at 2, 5, and 12 months of age (Zimmer et al., 1999). Care of the animals and conduction of all experiments followed the guidelines of European Communities Directive 86/609/EEC and the 1998 German Animal Protection Law regulating animal research.

### Eight-arm radial maze

**Figure 1:**
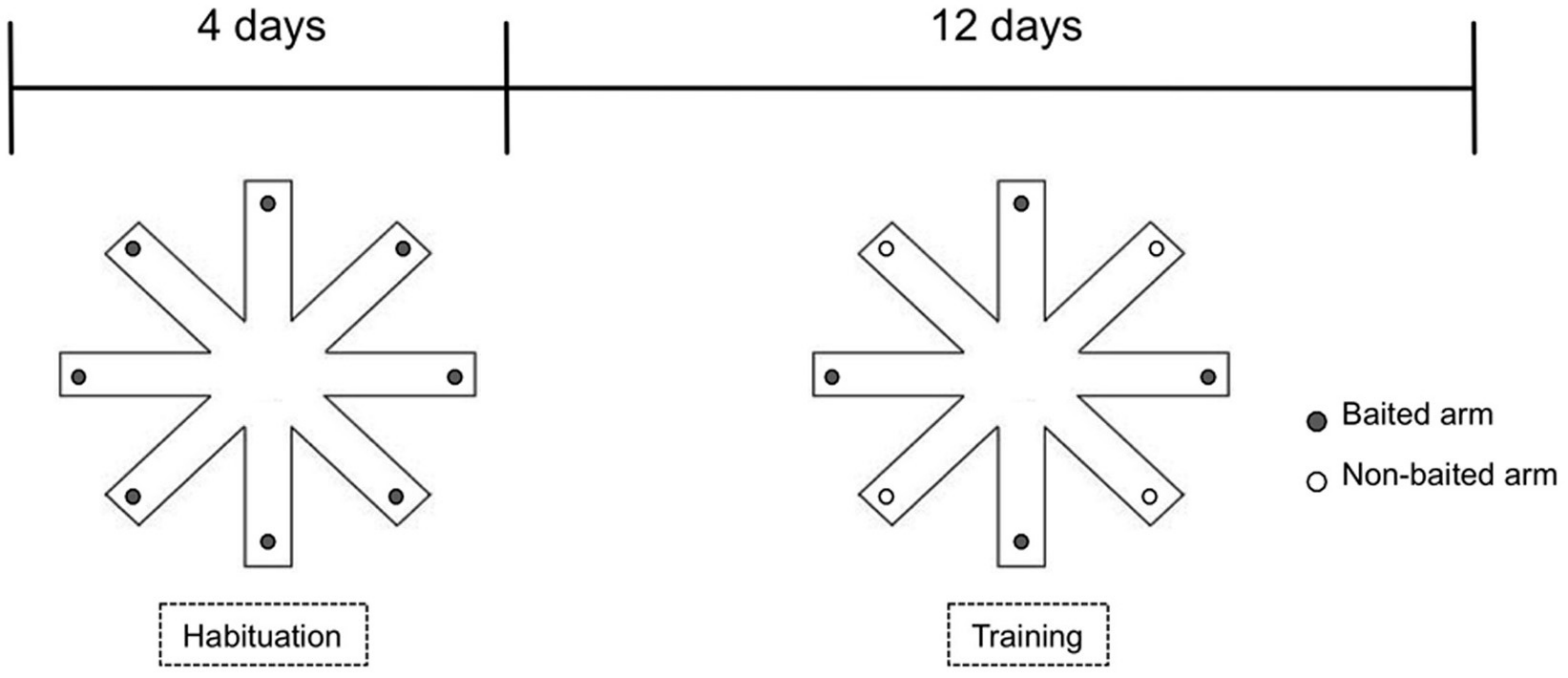
**Experimental setup of eight-arm radial maze test used for assessing reward—motivated learning and memory**.

The procedure was carried out with slight modifications as described previously (Heldt et al., 2007). Briefly, the eight-arm radial maze was constructed of white painted wood. It consisted a central platform (30 cm in diameter) with eight-arms (70 × 10 cm) surrounded by walls (10 cm in height) and placed in a sound and light isolated room with a camera on the ceiling. The behavior of the animals was followed by an experienced observer unaware of the genotype from an adjacent room through a monitor and evaluated using The Observer software (Noldus, Netherland). During the training, animals were single housed and maintained at 80–90% of their body weight by dietary restriction. To familiarize the animals to the radial maze, the mice received one daily habituation session for 4 days prior to training. During the habituation trial, all the eight-arms are baited with 1 food pellet, and the mouse was allowed to explore freely until it had taken all the pellets. After habituation, all mice were tested with 1 trial per day for 17 days (Figure 1). During the test trial, four randomly selected arms are baited with 1 pellet of food each; the baited arms are kept unchanged throughout the experiment. The mouse is allowed to move freely in the maze until it has collected the 4 pellets of food or until 10 min have elapsed, whichever occurs first. Entry into a non-baited arm was scored as a reference memory error, while re-entry into baited arms that had been previously visited was scored as a working memory error. The decrease in the number of arm entries per trial was used to indicate memory acquisition. The effect of deletion of CB1 receptor on the performance was evaluated separately in the age groups using Three-Way mixed ANOVA (between factors: genotype, sex; within factor: trial) followed by Bonferroni test.

## Results

**Figure 2:**
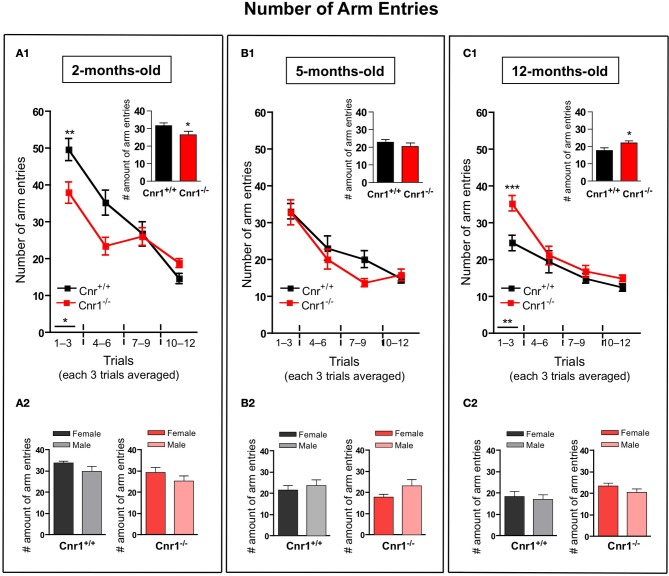
**Age-related changes in the number of arm entries in the eight-arm radial maze test between wild-type and Cnr1^−/−^ mice**. Each value represents the number of arm entries made until the mouse acquired all the rewards; mean ± S.E.M. **(A1,B1,C1)** on each of 4 blocks of 3 trial days. Graph inserts show the average of all trial days separately by age group. **(A2,B2,C2)** Show the main effects of genotype for males and females mice separately by each age group, mean ± S.E.M. for each sex and genotype averaged over the all trial days. The data were analyzed by Two-Way analyses of variance (ANOVA) followed by Bonferroni *post-hoc* test or One-Way analyses of variance (ANOVA) followed by Dunnett* post-hoc* test; ^*^p < 0.05; ^**^p < 0.01; ^***^p < 0.001. (*n* = 12 mice/group; 6 females and 6 males).

The total number of arm entries required to collected the food pellets or reach the cut-off time was significantly lower in 2-months-old Cnr1^−/−^ (genotype effect: *F*_(1, 264)_ = 8.28, *p* < 0.01) (Figure 2A1) and higher in 12-months-old Cnr1^−/−^ (genotype effect: *F*_(1, 264)_ = 13.93, *p* < 0.001) (Figure 2C1) mice compared to age-matched WT controls. However, the number of arm entries did not differ between the genotypes at 5 months of age (genotype effect: *F*_(1, 264)_ = 2.63, N.S.) (Figure 2B1). The sex of the animals did not influence the mean number of arm entries (Genotype × sex effect: 2-months-old: *F*_(1, 11)_ = 0.811; N.S.; 5-months-old: *F*_(1, 11)_ = 0.731; N.S.; 12-months-old: *F*_(1, 11)_ = 0.805; N.S.) (Figures 2A2,B2,C2, respectively).

**Figure 3:**
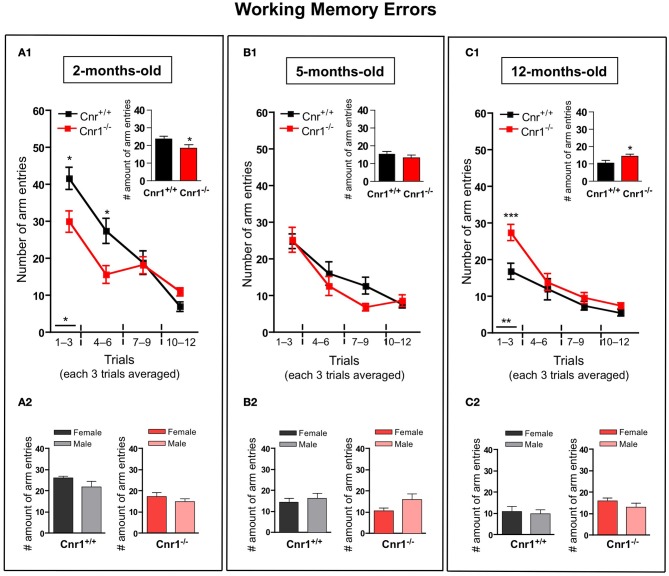
**Age-related changes in the working memory errors in the eight-arm radial maze test between wild-type and Cnr1^−/−^ mice**. Each value represents the working memory errors made until the mouse acquired all the rewards; mean ± S.E.M. **(A1,B1,C1)** on each of 4 blocks of 3 trial days. Graph inserts show the average of all trial days separately by age group. **(A2,B2,C2)** Show the main effects of genotype for males and females mice separately by age group, mean ± S.E.M. for each sex and genotype averaged over the all trial days. The data were analyzed by Two-Way analyses of variance (ANOVA) followed by Bonferroni *post-hoc* test or One-Way analyses of variance (ANOVA) followed by Dunnett *post-hoc* test; ^*^p < 0.05; ^**^p < 0.01; ^***^p < 0.001. (*n* = 12 mice/group; 6 females and 6 males).

**Figure 4:**
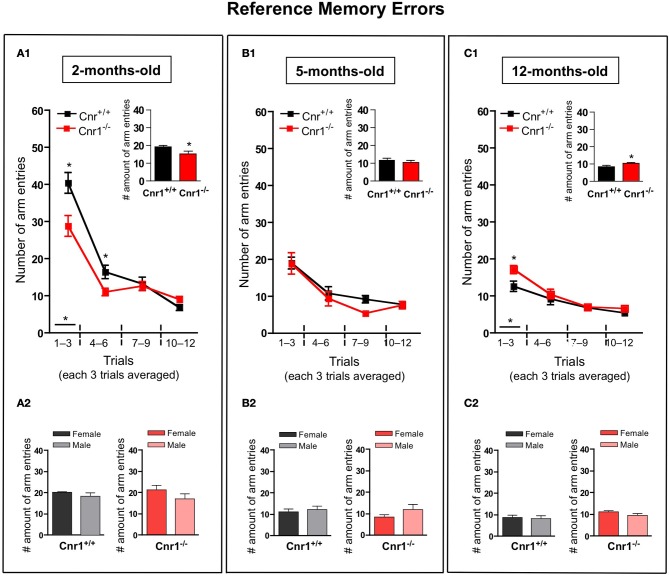
**Age-related changes in the reference memory errors in the eight-arm radial maze test between wild type and Cnr1^−/−^ mice**. Each value represents the reference memory errors made until the mouse acquired all the rewards; mean ± S.E.M. **(A1,B1,C1)** on each of 4 blocks of 3 trial days. Graph inserts show the averaged of all trial days separately by age group. **(A2,B2,C2)** show the main effects of genotype for males and females mice separately by age group, mean ± S.E.M. for each sex and genotype averaged over the all trial days The data were analyzed by Two-Way analyses of variance (ANOVA) followed by Bonferroni *post-hoc* test or One-Way analyses of variance (ANOVA) followed by Dunnett *post-hoc* test; ^*^p < 0.05 (*n* = 12 mice/group; 6 females and 6 males).

The number of working (Figure 3)—and reference (Figure 4)—memory errors generally showed similar results as the total number of arm entries. 2-months-old Cnr1^−/−^ animals showed significantly less errors (the number of working memory errors: genotype effect: *F*_(1, 264)_ = 8.30, *p* < 0.01; the number of reference memory errors: genotype effect: *F*_(1, 264)_ = 8.55, *p* < 0.01) while 12-months-old Cnr1^−/−^ animals made more errors (the number of working memory errors: genotype effect: *F*_(1, 264)_ = 14.39, *p* < 0.001; the number of reference memory errors: genotype effect: *F*_(1, 264)_ = 8.88, *p* < 0.01) during the task compared to age-matched WT littermates. There was no difference in both parameters at 5 months of age between Cnr1^−/−^ and Cnr1^+/+^ mice (the number of working memory errors: genotype effect: *F*_(1, 264)_ = 2.41, N.S.; the number of reference memory errors: genotype effect: *F*_(1, 264)_ = 2.11, N.S.). The sex of the animals also did not influence the number of working memory errors (Genotype × sex effect: 2-months-old: *F*_(1, 11)_ = 0.829; N.S.; 5-months-old: *F*_(1, 11)_ = 0.778; N.S.; 12-months-old: *F*_(1, 11)_ = 0.833; N.S.) or the number of reference errors (Genotype × sex effect: 2-months-old: *F*_(1, 11)_ = 1.209; N.S.; 5-months-old: *F*_(1, 11)_ = 0.678; N.S.; 12-months-old: *F*_(1, 11)_ = 0.245; N.S.).

**Figure 5:**
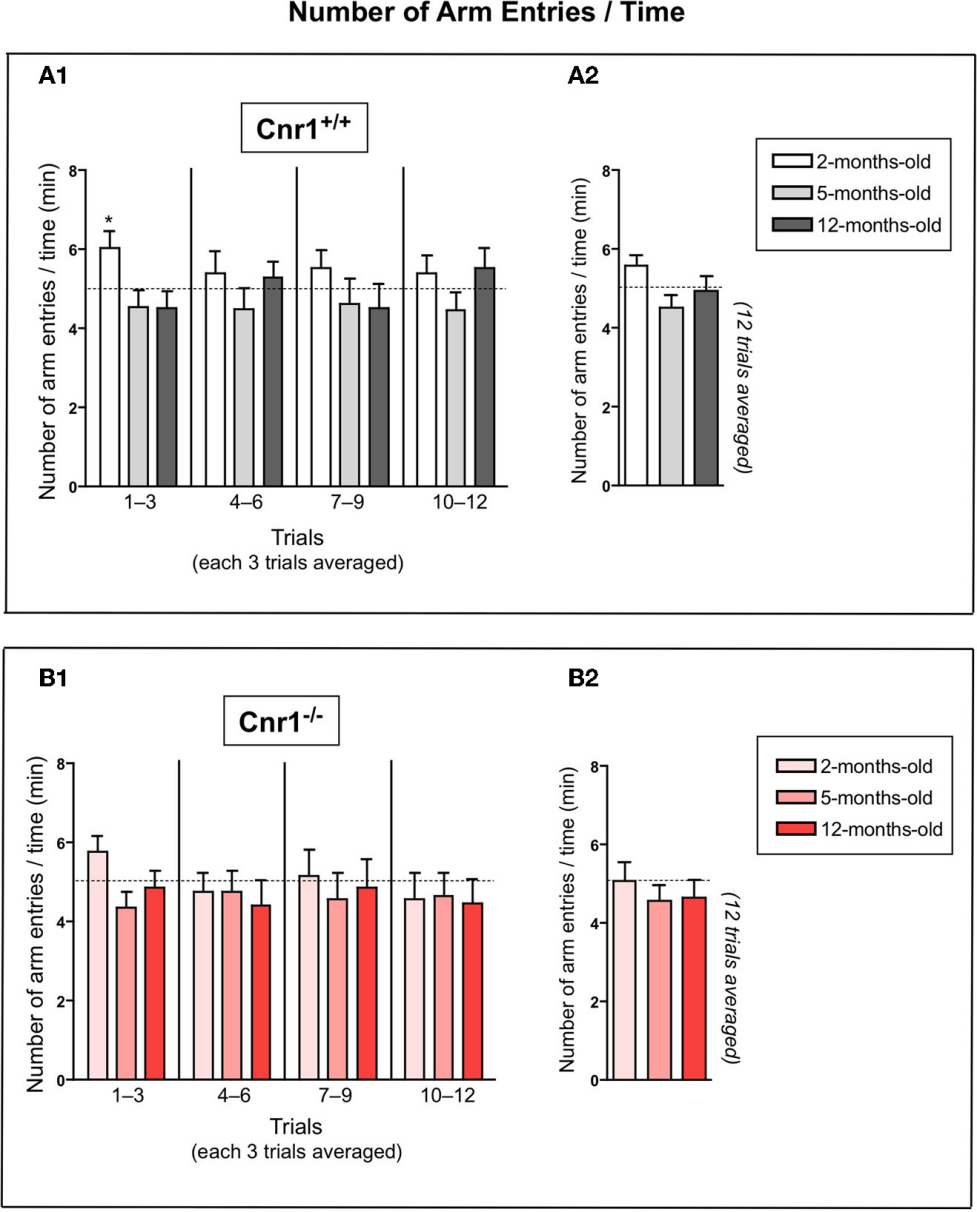
**The ratio between number of arm entries and time spent until the mouse acquired all the rewards in the eight-arm radial maze test**. There is a significant age effect on day 1–3 **(A1,B1)**, 2-months-old mice initially made more errors in a shorter period but quickly learned the task. The data were analyzed by One-Way analyses of variance (ANOVA) followed by Dunnett *post-hoc* test; ^*^p < 0.05 (*n* = 12 mice/group; 6 females and 6 males). No significant age effect for the 12 trials average of all trial days by each genotype **(A2,B2)** using One-Way ANOVA.

It was previously suggested that high levels of vigorous and impulsive activity, which is typical in young rodents, could interfere with the performance in learning paradigms (Dodart et al., 1999; Ward et al., 1999). This phenomenon was also present in our study: in the first phase of the experiment at days 1–3, the speed of the young animals expressed as number of arm entries within a minute was significantly higher in 2-months-old mice compared to the other age-groups (Figure 5A1). A similar, but not significant tendency was observed in Cnr1^−/−^ mice (Figure 5B1). However, there was no difference in the speed between the age groups when we evaluated each trial together (Figures 5A2,B2).

In the eight-arm radial maze test of appetitive conditioning, Cnr1^−/−^ mice begin to show impairments at 12-months-old compared to age-matched WT controls that is similar, albeit at a slightly older age, to results using an aversive conditioning paradigm (Albayram et al., 2011).

## Discussion

Our present study using the radial arm maze shows that the life-long deletion of CB1 receptors has opposite effects on young and old animals, as also found in the Morris water maze test (Albayram et al., 2011). Thus, our result suggests that age-related effect of CB1 receptor activity is the same on reward and aversive-driven models of learning.

Cannabinoid signaling is involved in reward signaling and influences stress reactivity. Genetic deletion or pharmacological blockade of CB1 receptor decreases sensitivity to reward (Xi et al., 2008; Oleson et al., 2012) but exacerbates stress reactivity (Griebel et al., 2005; Riebe and Wotjak, 2011; Gunduz-Cinar et al., 2012). These data together with findings showing that cannabinoid signaling differently affects memories with positive and negative emotional valence (Marsicano et al., 2002; Laviolette and Grace, 2006; Terzian et al., 2011) strongly suggests that the role and activity of the cannabinoid system differs in reward and fear-related neuronal pathways and brain areas. In aging, however, our study now suggests that the effect of enhanced neuronal loss (Bilkei-Gorzo et al., 2005) and increased neuroinflammation in the hippocampus of Cnr1^−/−^ animals (Albayram et al., 2011) generally impairs hippocampal learning and memory affecting both reward and aversion-driven spatial learning.

Previous studies have also shown that THC treatment impairs performance in the radial arm maze test (Lichtman and Martin, 1996), the effect of which was attenuated by the systemic (Lichtman and Martin, 1996) or intrahippocampal (Wise et al., 2009) injection of the CB1 receptor antagonist SR141716A. Pharmacological blockade of the CB1 receptors produced an opposite effect: mice injected with only SR141716A made fewer errors in the acquisition phase (Lichtman, 2000) and the CB1 receptor antagonist CE improved consolidation of spatial memory (Wise et al., 2008). Nevertheless, our study now proves that lack of CB1 receptors in Cnr1^−/−^ mice leads to similar improvement in the performance of young mice in the radial arm maze test as pharmacological blockade of CB1 receptors. Although experimental evidence about the effect of CB1 receptor antagonist on old animals in the eight-arm maze test is lacking, it is unlikely that long-term pharmacological treatment can equate life-long deletion of the CB1 receptors and thus long-term antagonist treatment would be not feasible (Kunos and Tam, 2011).

An interesting aspect of our work is that males and females showed a similar performance although earlier studies using the Morris water maze paradigm suggested that the spatial learning abilities differs between the sexes (Clinton et al., 2007; Hebda-Bauer et al., 2007). A possible reason for this discrepancy is that the test situation is highly aversive in the Morris water maze test (Thorsell et al., 2000), whereas the eight-arm radial maze test condition is much less stressful, since it is based on the innate drive of the to explore the environment and seeking for food (Lehmann et al., 2010). Environmental stress influences memory formation differently in males and females (Andreano and Cahill, 2009; Oldehinkel and Bouma, 2011). Epidemiological studies in humans (Luine, 2002; Richardson and VanderKaay Tomasulo, 2011) and experimental studies in rodents (Sanders et al., 2011; ter Horst et al., 2011) together suggest that sexual dimorphisms in the hypothalamic–pituitary–adrenal axis response are responsible for these effects. Apparently, females are more sensitive to the effect of stress due to fluctuations in gonadal hormone levels (Paris et al., 2011). Indeed, recent studies have revealed that gonadal hormones have a beneficial effect on memory formation in females under stressful conditions (Streeten et al., 1984). However, the stress response is not consistently different between the sexes in adolescents (Stoney et al., 1987; Gallucci et al., 1993; Greenspan et al., 1993), whereas this effect is present when testing adults (Peskind et al., 1995; Thorsell et al., 2000). Thus, we propose that the difference in the learning ability between the sexes in the Morris water maze test is based on their difference in stress sensitivity. This phenomenon was probably not present in the less aversive model of spatial learning—in the eight-arm maze paradigm—and therefore did not influence the performance of the animals.

Multiple lines of evidence prove that more emotional events are remembered better than non-emotional ones (Hamann, 2001; LaBar and Cabeza, 2006). Although the input of emotions on memories is also present in the old, especially when emotional arousal is high (da Silva et al., 2007; Kensinger, 2008; Ramirez-Lugo et al., 2009), its effect becomes less and less pronounced during ageing (Comblain et al., 2004; Bressler et al., 2010). Our results now suggest that the decreasing CB1 receptor signaling during ageing (Wang et al., 2003; Canas et al., 2009) does not contribute to this effect.

### Conflict of interest statement

The authors declare that the research was conducted in the absence of any commercial or financial relationships that could be construed as a potential conflict of interest.
